# Encapsulating Peritoneal Sclerosis in Long-Termed Peritoneal Dialysis Patients

**DOI:** 10.1155/2018/8250589

**Published:** 2018-11-13

**Authors:** Heng-Jung Hsu, Shih-Ying Yang, I-Wen Wu, Kuang-Hung Hsu, Chiao-Yin Sun, Chun-Yu Chen, Chin-Chan Lee

**Affiliations:** ^1^Division of Nephrology, Chang Gung Memorial Hospital, Keelung, Taiwan; ^2^College of Medicine, Chang Gung University, Taoyuan, Taiwan; ^3^The Graduate Institute of Clinical Medical Sciences, Chang Gung University Medical College, Taoyuan School of Medicine, Taiwan; ^4^Community Medicine Research Center, Keelung Chang Gung Memorial Hospital, Keelung, Taiwan; ^5^Healthy Aging Research Center, Chang Gung University, Taoyuan, Taiwan; ^6^Laboratory for Epidemiology, Department of Health Care Management, Chang Gung University, Taoyuan, Taiwan; ^7^Department of Emergency Medicine, Chang Gung Memorial Hospital, Taoyuan, Taiwan; ^8^Department of Urology, Chang Gung Memorial Hospital, Taoyuan, Taiwan

## Abstract

**Background:**

Encapsulating peritoneal sclerosis (EPS) is a rare but serious clinical complication of long-term peritoneal dialysis (PD) patients with high mortality. The purpose of this study was to assess the clinical characteristics of patients with EPS and to search for possible factors useful for EPS prevention and early diagnosis.

**Method:**

This retrospective study was performed in a single dialysis center in Taiwan between August 1990 and April 2014. Overall, a total of 565 patients were included and the medical records of those patients who had developed EPS (EPS group) and those who had not developed EPS (control group) were collected. We compared several factors between these two groups.

**Result:**

In the univariate analysis, EPS was significantly associated with a change of transport state (Delta 2) (*p* = 0.007), duration of PD (*p* < 0.001), duration of peritonitis treatment (*p* = 0.001), number of peritonitis episodes (*p* = 0.002), and fungus related peritonitis (*p* = 0.031). After multivariate logistic model analysis, we found that only the duration of PD was independently significantly associated with EPS (*p* = 0.034). In addition, we used the ROC curve and found that a duration of peritoneal dialysis of about 8.4 years is the best cut-off point to predict EPS occurrence.

**Conclusion:**

In this study, long-termed PD duration is the only strong independent risk factor for EPS development. Total peritonitis times, total peritonitis treatment duration, and marked increased peritoneal D/P_cr_ ratio were also significantly associated with the duration of PD.

## 1. Background

Chronic Kidney Disease (CKD) is defined as a decrease in the estimated glomerular filtration rate, an increase in urinary protein excretion, or both. The prevalence of CKD is increasing day by day and is a worldwide public health problem. The prevalence rate of CKD in the world is around 8–16% [1]. The reasons of high prevalence rate of CKD include the aging of general populations, rising prevalence of diabetes mellitus (DM) and hypertension, and reducing death of organs failure patients in stroke and myocardial infarction due to the development of treatments [2].

The incidence of end stage renal disease (ESRD) in Taiwan is the highest in the world according to the United States Renal Data System (USRDS) 2015 report (2013 registration data) and far higher than European countries, the United States and Japan [3]. Furthermore, the prevalence rate of ESRD in Taiwan is also high compared to the rest of the world. Therefore, the costs associated with managing ESRD are very high in our country. In addition to the huge cost of ESRD management, patients who suffer from ESRD also experience a decreased quality of life [4, 5] and lifespan [6].

Peritoneal dialysis (PD) is one of the options for patients with ESRD though the proportion of its use is falling in many developing countries [7]. The number of patients treated with peritoneal dialysis rose worldwide from 1997 to 2008, with a 2.5-fold increase in the prevalence of peritoneal dialysis patients in developing countries and representing 11% of the global dialysis population [7]. PD has comparable mortality risks but is significantly less costly in most parts of the world [8–10]. The primary advantage is the ability to undertake treatment without having to visit a medical facility. In addition, PD might work better in preserving remaining kidney function and can provide a better quality of life than hemodialysis. Despite the advantages associated with this modality, one rare but catastrophic risk is the development of encapsulating peritoneal sclerosis (EPS). EPS is a rare but serious clinical complication of long-term PD patients with high mortality. It is characterized by a progressive thickening of the peritoneum and calcification to encase the intestinal tract into a cocoon-like form, causing partial or complete obstruction of the intestinal tract and eventually leading to malnutrition and sepsis [11].

The incidence of EPS in PD patient has been reported to be 0.7% to 7.3% [12], and the rate appears to be higher in patients receiving long-term PD treatment. The development of EPS might be directly proportional to the duration of PD treatment [13–16]. Current International Society for peritoneal dialysis (ISPD) guidelines for EPS showed the risk for developing EPS is considered very low during the first 3 years of PD treatment and low for patients with less than 5 years on PD [17]. A Japanese study showed that the incidence of EPS was 0.3%, 0.6%, and 2.3%, respectively, in the patients in the third, fifth, and eighth year of the peritoneal dialysis. The prognosis of EPS is very poor with mortality rates from 25% to 55% in the first year after diagnosis [15].

In addition to EPS not being easy to diagnose by noninvasive methods, there also have been no definitive treatments for EPS established to date. The pathophysiology of EPS is still largely unknown. Several factors, such as the duration of PD, age at first PD, PD-associated peritonitis, high dialysate/plasma creatinine ratio (D/P_cr_), and exposure to icodextrin, high-glucose dialysate, have been suggested to predispose patient to developing EPS. The purpose of this study was to assess the clinical characteristics of patients with EPS and to search for possible factors useful for EPS prevention and early diagnosis.

## 2. Materials and Methods

### 2.1. Study Population and Design

Our study was conducted at the single dialysis center of the Chang Gung Memorial Hospital (Keelung, Taiwan). We retrospectively reviewed records of all patients who started PD at the hospital between August 1990 and April 2014. We separated the patients into two groups for the purpose of analysis: those who had developed EPS (EPS group) and those who had no documentary evidence of EPS (control group). This study was approved by the Institutional Review Board (IRB) of the Chang Gung Memorial Hospital (201701573B0).

EPS was diagnosed according to the criteria developed by the International Society for peritoneal dialysis (ISPD) Ad Hoc Committee on EPS in 2001 [11], which states that EPS needs to be evaluated in three parts: (1) the clinical diagnosis, (2) the radiologic diagnosis, and (3) the pathologic diagnosis. We made the diagnosis of our PD patients with EPS by clinical presentations, including poor appetite, nausea, vomiting, abdominal pain, malnutrition, and unresolved peritonitis. The radiology study of CT scans revealed thickening intestinal walls and peritoneal membranes, increased density of mesenteric fat, adherent dilated bowel loops, and loculated ascites. All EPS patients that received a diagnostic laparoscopy or laparotomy found diffuse fibrin coating in the visceral and parietal peritoneum, turbid ascites and the presence of an abdominal cocoon. Pathology showed decreased cellularity, fibrin deposits and a complete denudation of the mesothelial cell layer with fibrin exudations.

Overall, a total of 559 patients were included in the control group based on their medical records, compared to 6 patients in the EPS group. We compared several factors between the two groups, including age of dialysis, gender, comorbidity of DM, the latest laboratory data in routine examinations, duration of PD, peritoneal transport states, changes in peritoneal membrane transport states, number of peritonitis episodes, duration of peritonitis treatments, and fungus related peritonitis. We defined a change of peritoneal membrane transport state by comparing the first and last PET during peritoneal dialysis. The change of the PET exams between the first and the last PET exam from low to lower average, lower average to high average, or high average to high is defined as Delta 1; the PET exam from low to high average or lower average to high is defined as Delta 2.

### 2.2. Statistical Analysis

Continuous variables were presented as means the ± the standard deviations (SD). For normally distributed continuous variables, a two-tailed Student's unpaired t test was employed to evaluate the differences between the means. Group differences of categorical variables were determined via either the chi-square or Fisher's exact test. A Pearson's correlation test was used to examine the relationships between the duration of PD and other variables (include the number of peritonitis episodes and duration of peritonitis treatment). To evaluate the impact of PD-related factors on the risk of developing EPS during long-term PD treatment, univariate and multivariate logistic regression models were applied. A receiver operating characteristic (ROC) curve was used to find the optimal cut-off points, which represented the duration of PD to predict EPS occurrence. All statistical tests were two tailed, and a* p* value of < 0.05 was considered statistically significant. All analyses were performed using SPSS software version 17.0 for Windows (SPSS; Chicago, IL, USA), a commercially available statistics software package.

## 3. Results

In the period of August 1990 to April 2014, there were six cases of EPS occurring in our hospital. All cases of EPS were diagnosed by laparoscopy, and the prevalence of EPS in our center was about 1.07%. Two patients were diagnosed as EPS whilst on HD, one patient was diagnosed as EPS past kidney transplantation, whereas other three patients were diagnosed as EPS whilst on PD. All patients with EPS shifted to hemodialysis modality and two patients died due to EPS and related infection. In our study patients, there were 319 males (56.5%) with the mean age on dialysis around 53.67 ± 17.63 years old (Table 1). Around 39.5% of cases had the primary cause of DM. The mean duration of PD was 3.1 ± 2.8 years. According to the results of the initial peritoneal equilibration test (PET) exam, 269 patients (47.6%) were classified as “high transport”, 183 patients (32.4%) as “high average”, 98 patients (17.3%) as “low average”, and 15 patients (2.7%) as “low transport”. We found that the age of PD patients developing EPS was similar to the patients without EPS (54.3 ± 18.27* versus* 53.7 ± 17.60 years old,* p* = 0.935). The rates of gender, DM, initial and last peritoneal transport state were similar between the two groups. Compared with the control group, changes of the peritoneal transport state Delta 2 (*p *= 0.044) and the duration of PD (*p* < 0.001) were significantly associated with EPS.

For the laboratory exam, there were no significant differences between the two groups in the inflammatory or nutrition markers such as white blood count (WBC), ferritin, albumin, hemoglobin or cholesterol (Table 2). We also collected information about the episodes of peritonitis and the duration of peritonitis treatment. We found that the episodes of peritonitis were significantly higher among EPS patients than non-EPS patients (EPS* versus* Non-EPS: 3.17 ± 2.86* versus* 0.95 ± 1.46 times,* p* < 0.001). The duration of peritonitis treatment was significantly longer in EPS patients than non-EPS patients (EPS* versus* Non-EPS: 41.33 ± 44.72* versus* 11.20 ± 18.71 days,* p* < 0.001). The incidence of fungus related peritonitis was significantly higher in EPS than non-EPS patients (EPS* versus* Non-EPS: 33.3%* versus* 7.0%, times,* p* = 0.013). About those patients suffered from fungus related peritonitis, all of the patients received the surgery of PD catheter removal during the antifungus treatment. And eight of those forty-one patients received the PD modality after the antifungus treatment course about 3 months. Others continuously received the HD modality after the antifungus treatment course.

In our initial analysis, we found that the duration of PD, changes of transport state (Delta 2), number of peritonitis episodes, peritonitis treatment duration, and fungus related peritonitis were significantly associated with EPS. Therefore, we then used univariate logistic regression followed by multivariate logistic regression to examine the independent factors associated with EPS (Table 3). In the univariate analysis, EPS was significantly associated with change of transport state Delta 2 (*p* = 0.007), duration of PD (*p* < 0.001), duration of peritonitis treatment (*p* = 0.001), number of peritonitis episodes (*p* = 0.002), and fungus related peritonitis (*p* = 0.031). After multivariate logistic model analysis, we found that only the duration of PD was independently significantly associated with EPS (*p* = 0.034).

In our study, we found that the duration of PD is the only factor independently associated with EPS whereas the total number of peritonitis times and the total peritonitis treatment duration lost their significance after multivariate logistic regression. In order to evaluate the relationship between the duration of PD with total peritonitis times and peritonitis treatment duration, we analyzed the correlation between them. We found that the duration of PD was significantly positively correlated with total peritonitis times (correlation factor: 0.351,* p *< 0.001) and total peritonitis treatment duration (correlation factor: 0.337,* p* < 0.001). Furthermore, we evaluated the association between the duration of PD with the presence of fungus peritonitis and the change to transport state Delta 2. We found that patients with the presence of fungus peritonitis were not significantly associated with the duration of PD (3.515 ± 2.74* versus* 3.066 ± 2.81 years,* p* = 0.324). However, PD patients with changes of transport-Delta 2 had significantly longer durations of peritoneal dialysis (7.22 ± 4.22 v*ersus* 2.72 ± 2.65 years,* p* < 0.001).

**Figure 1:**
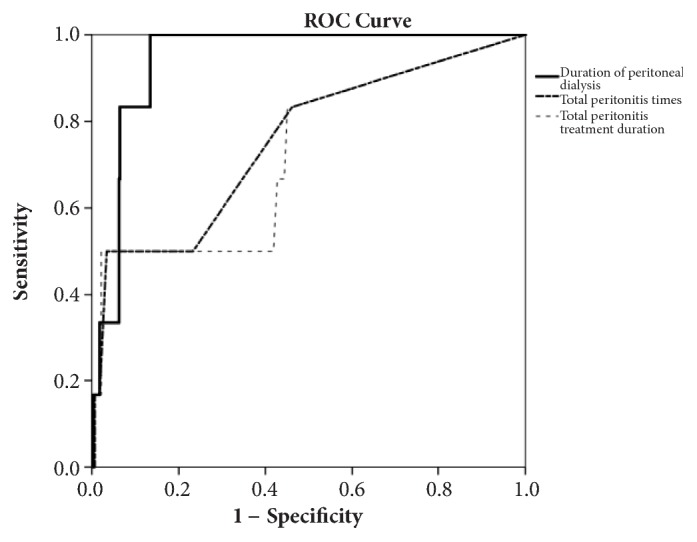
The receiver operating characteristic (ROC) curves for total peritonitis times, total peritonitis duration, and the duration of PD in predicting occurrence of encapsulating peritonitis sclerosis on CKD patients on peritoneal dialysis.

The duration of PD is an important factor associated with EPS in our study. Therefore, it is very interesting to note the high possibility that the duration of peritoneal dialysis is associated with EPS occurrence. Consequently, we used the ROC curve to evaluate the duration of PD and EPS occurrence (Figure 1). We found that the area under the curve of the duration of dialysis was significantly higher than total number of peritonitis times and also the total peritonitis treatment duration (AUC of duration of PD* versus* total number of peritonitis times* versus* total peritonitis treatment duration: 0.942* versus* 0.742* versus* 0.725,* p* < 0.001). We also used the ROC curve to find a point with better sensitivity and specificity (sensitivity: 83.3%, specificity: 93.6%). We found that the duration of peritoneal dialysis for 8.4 years is the best cut-off point to predict EPS occurrence.

## 4. Discussion

This study was conducted to clarify the clinical risk factors related to the presence of EPS in 565 patients who had undergone peritoneal dialysis treatment. There were significant differences in the duration of PD, number of peritonitis episodes, peritonitis treatment duration, fungus related peritonitis, and increased peritoneal D/P_cr_ ratio (Delta 2) during PD between the patients who developed EPS and those who did not. Multivariate analysis showed that only the PD duration was significantly associated with the presence of EPS. The incidence of EPS would rise in patient receiving PD for longer periods.

In our study, we found 6 patients with EPS among the 565 PD patients. The overall prevalence of EPS was 1.07% in the 24 -year period. This is similar to the rates reported in previous Japanese and American studies (1.1% and 1.2%) [18, 19]. However, recent studies have found that the prevalence of EPS is increasing compared to previous reports. An Italian study revealed an EPS prevalence of 2.8% [20] and an Iranian center showed the prevalence of EPS at 2.6% [21]. The rise in the prevalence of EPS in recent studies reflected both a longer duration of PD exposure and higher awareness of EPS. In our study, the incidence of EPS was 6.7% among those who remained on PD for more than 6 years. The observed risk of EPS after 5 years of PD has been reported to vary from 2.1% in Japan [13] to 6.4% in Australia [12] and 8.1% in Scotland [22]. It is well documented that many patients develop EPS when switched to HD [23]. These patients who changing modality to HD are difficult to follow up and some of them may develop EPS later. The incidence of EPS by reviewing PD patients or shifting to our hospital HD center or transplantation may be underestimated in our study.

Nakayama M. et al. reported that increased peritoneal transport state is a risk factor for EPS developing after long-term peritoneal dialysis [24–26]. Prolonged PD therapy leads to morphology changes in the peritoneal membrane. These changes would ultimately trigger peritoneal sclerosis [27]. Therefore, we try to elucidate if an increased peritoneal D/P_cr_ ratio might allow for early detection of EPS development. In the present study, a marked increased peritoneal D/Pcr ratio (Delta 2) during PD was revealed to be significantly higher in the EPS group as compared to the non-EPS group. However, an increased peritoneal D/Pcr ratio (Delta 2) was not identified as significant after multivariate analysis.

A number of studies have emphasized the association between peritonitis related factors and EPS development [23, 28–30]. In our study, the total peritonitis times, duration of peritonitis treatment, and fungus peritonitis were significantly different between the EPS and non-EPS groups. However, neither of these factors was associated with EPS in multivariate analysis. In an EPS study from the Netherlands, Korte et al. also could not find a relationship between peritonitis and EPS development [31]. Similarly, Johnson et al. showed no association between peritonitis frequency and EPS risk [15]. Recently, in Iran, a study found both peritonitis rate and total peritonitis episodes were not significantly different between EPS and control groups in regression analysis [21]. In our study, we found that PD duration is the only strong independent factor associated with EPS. Additionally, we also found that PD duration was highly correlated with total peritonitis times and total peritonitis treatment duration. Furthermore, PD duration was also significantly associated with increased peritoneal D/Pcr ratio (Delta 2).

We used an ROC curve to find that a PD duration of 8.4 years was a very sensitive and specific spot to predict EPS development in the patients in this study. Yamamoto et al. reported that their ROC analysis showed that the PD duration to predict EPS development would be 115.2 months (9.6 years) [28]. Among the numerous clinical studies, long-term PD duration has been consistently demonstrated to be a strong risk factor for the development of EPS [12, 20, 22, 31–34]. But, there is no consensus on the planned discontinuation of peritoneal dialysis. According to these data and also our core findings, planned discontinuation of PD after 8 years to prevent EPS development might be a feasible strategy for high risk patient such as peritonitis, inadequate ultrafiltration, high transport state, and frequent use of high-dextrose solution.

In conclusion, our study was conducted at a single dialysis center in Taiwan and compared numerous clinical parameters between the chronic PD patients who did and did not develop EPS in order to identify independent predictors of EPS. We used univariate logistic regression followed by multivariate logistic regression and found that long-term PD duration is the only strong independent risk factor for EPS development. Total peritonitis times, total peritonitis treatment duration, and marked increased peritoneal D/P_cr_ ratio (Delta 2) were significantly associated with the duration of PD. The advantage of this study is a relatively large number of patients in the control group and the uniform criteria used for diagnosis of EPS. We also clarified the interaction between PD duration and peritonitis and increased peritoneal D/P_cr_ ratio. However, the present study is limited by its retrospective nature, very small numbers of cases of EPS, and lacks data collection for dialysate composition, severity of comorbidities, treatment of EPS, and overall life expectancy of study patients. However, this study found the most likely period of EPS development—8.4 years—and also explains the nature of the EPS development based on the factor of PD duration rather than peritonitis times or peritonitis durations. Because there is still no optimal treatment for EPS and the prognosis of EPS is very poor, we think this result can help PD patients avoid the development of EPS and remain alive.

## Figures and Tables

**Table 1:** Demographics and clinical characteristics of the 565 patients in the study.

	All patients (N = 565)	EPS group (N = 6)	Control group ( N = 559)	P value
**Age on dialysis **	53.67 ± 17.63	54.3 ±18.27	53.7 ±17.60	0.935
**Gender, male **	319 (56.5%)	3(50.0%)	316(56.5%)	0.748
**Diabetes mellitus (**%**)**	223 (39.5%)	0(0%)	223 (39.9%)	0.086
**Transport state of PET (initial)**				0.21
High^a^	269 (47.6%)	1(16.7%)	268(48.1%)	
High Average^a^	183 (32.4%)	2 (33.3%)	181(32.5%)	
Low Average^a^	98 (17.3%)	3(50.0%)	95(16.9%)	
Low^a^	15 (2.7%)	0(0.0%)	15(2.6%)	
**Transport state of PET(last)**				0.51
High	82 (14.5%)	2(33.3%)	80(14.3%)	
High Average	264 (46.7%)	3(50.0%)	261(46.7%)	
Low Average	194 (34.3%)	1(16.7%)	193(34.6%)	
Low	25 (4.5%)	0 (0.0%)	25(4.5%)	
**Change of transport state** ^**b**^				
Delta 1^b^	292 (51.7%)	2( 33.3%)	290(51.9%)	0.279
Delta 2^b^	111 (19.6%)	2(33.3%)	109(19.5%)	0.044*∗*
**Duration of PD (years)**	3.1 ± 2.8	9.05±2.21	3.05±2.73	<0.001*∗*

EPS = encapsulating peritoneal sclerosis.

PET = peritoneal equilibration test.

PD = peritoneal dialysis.

D/P ratio Cr = dialysate-to-plasma concentration ratio for creatinine.

^a^High defined as D/P ratio Cr >0.80, High average as D/P ratio Cr between 0.65 and 0.80, Low average as D/P ratio Cr between 0.55 and 0.64, and Low as D/P ratio Cr < 0.55.

^b^The change of the PET exam between the first and the last PET exam from low to lower average, lower average to high average, or high average to high is defined as Delta 1; PET exam from low to high average or lower average to high defined as Delta 2.

**Table 2:** Peritonitis related factors and laboratory examination of the patients.

	All ( N=565)	EPS group (N = 6)	Control group (N = 559)	P value
**Peritonitis related factors**				
Peritonitis history (%)	263 (46.5%)	5 (83.3%)	258(46.2%)	0.069
Number of Peritonitis episodes	0.97 ± 1.45	3.17 ± 2.86	0.95 ± 1.46	<0.001*∗*
Peritonitis treatment duration (days)	11.52 ± 19.33	41.33 ± 44.72	11.20 ± 18.71	<0.001*∗*
Fungus related peritonitis (%)	41 (7.25%)	2(33.3%)	39(7.0%)	0.013*∗*
**Lab**				
Albumin (mg/dL)	3.20 ± 0.75	3.30 ± 0.80	3.20 ± 0.75	0.757
Creatinine (mg/dL)	10.23 ± 3.93	13.02 ± 4.80	10.20 ± 3.91	0.083
Hb (g/dL)	9.92 ± 1.73	9.28 ± 2.03	9.93 ± 1.73	0.361
WBC (1000uL)	9.08 ± 4.87	8.03 ± 1.53	9.10 ± 4.90	0.596
Ferritin (ng/mL)	675.2 ± 1244.2	653.2 ± 581.3	675.4 ± 1250	0.965
Calcium (mg/dL)	13.50 ± 9.34	9.80 ± 0.57	9.34 ± 1.13	0.319
Phophoate (mg/dL)	5.02 ± 1.70	5.13 ± 1.40	5.03 ± 1.71	0.878
iPTH (pg/mL)	273.7 ± 420.3	348.7 ± 344.8	272.8 ± 421.2	0.661
Potassium (meq/L)	3.79 ± 0.87	5.13 ± 1.40	5.03 ± 1.71	0.895
Uric acid ( mg/dL)	6.44 ± 1.45	5.63 ± 1.35	6.45 ± 1.48	0.179
Cholesterol (mg/dL)	194 ± 64.82	210 ± 58.56	194 ± 64.96	0.566
Triglyceride (mg/dL)	217 ± 256.19	267 ± 122.70	216 ± 257.28	0.629

EPS = encapsulating peritoneal sclerosis.

WBC = white blood cell.

Hb = hemoglobin.

iPTH = intact parathyroid hormone.

**Table 3:** Univariate and multivariate logistic models of risk factors associated with EPS.

	Univariate odd ratio	95% confidence interval	P value	Multivariate odd ratio	95% confidence interval	P value
Change of transport state (Delta 2)	0.133	0.030-0.583	0.007*∗*			
Duration of PD (years)	1.561	1.251-1.947	<0.001*∗*	1.42	1.026-1.964	0.034*∗*
Number of peritonitis episodes	1.532	1.168-2.008	0.002*∗*			
Peritonitis treatment duration (days)	1.033	1.013-1.054	0.001*∗*			
Fungus related peritonitis	6.667	1.184-37.540	0.031*∗*			

EPS = encapsulating peritoneal sclerosis.

PD = peritoneal dialysis.

## Data Availability

The data used to support the findings of this study are included within the article.
